# *In* *vivo* biomechanics after total hip arthroplasty: a state-of-the-art systematic review

**DOI:** 10.1530/EOR-2026-0003

**Published:** 2026-07-01

**Authors:** Vasileios Giannatos, Ioannis Siozos, Irini Tatani, Andreas Panagopoulos, Panagiotis Antzoulas, Sofia Xergia

**Affiliations:** ^1^ Orthopedics Department, University Hospital of Patras, Patras, Greece; ^2^ Physical Therapy Department, University of Patras, Patras, Greece

**Keywords:** total hip arthroplasty, THA, gait, biomechanics, EMG, dual fluoroscopy, motion analysis, force plates

## Abstract

**Purpose:**

- This systematic review aimed to synthesize current literature on *in vivo* biomechanics following total hip arthroplasty, emphasizing objective laboratory assessments and surgical modifiers to address persistent functional deficits despite favorable clinical outcomes.

**Methods:**

- A PRISMA-guided systematic review was conducted across seven databases (PROSPERO: CRD42024550457). Eligible studies included human subjects with objective postoperative biomechanical assessments (e.g. 3D motion analysis, force plates, dual fluoroscopy) compared against healthy or contralateral limbs. Data on spatiotemporal parameters, kinematics, kinetics, and surgical subgroups were extracted from 50 included studies.

**Results:**

- Key findings revealed persistent reductions in hip joint power, gait speed, and stride length, along with joint loading asymmetries >1 year post-operatively. While the direct anterior approach showed faster early gait normalization, the surgical approach had a minimal long-term impact. Conversely, restoring femoral offset and anteversion was strongly associated with improved kinetics. Older patients exhibited greater compensatory trunk movements.

**Conclusion:**

- The choice of surgical approach has a limited influence on long-term biomechanical restoration. Surgeons should instead prioritize offset restoration, implant choice, and functional component orientation, as static imaging fails to capture dynamic edge-loading. Future rehabilitation must target persistent kinetic asymmetries that standard clinical scores overlook.

## Introduction

Total hip arthroplasty (THA) has evolved significantly over the past several decades and has become one of the most successful and commonly performed procedures in medicine (1). Initially introduced in the 1960s, THA has undergone continuous refinement of implant materials, fixation techniques, and surgical approaches (2). Advances in component design and minimally invasive techniques have aimed to reduce surgical trauma, expedite recovery, and improve functional outcomes (2, 3).

Despite these improvements, growing evidence suggests that patients may continue to experience altered gait patterns and joint loading after surgery despite these being coupled with excellent clinical outcomes (4, 5). Known gait deficits post-operatively after THA include reduced step/stride length, reduced walking speed, reduced single limb support time, and peak hip extension due to antalgic gait alteration adapted pre-operatively in order to unload the pathologic hip joint, while abductor moments seem to be restored at 1-year (4, 5). Despite abductor moments restoration, abductor strength remains reduced compared to pre-operatively, but improves even at 2 years post-operatively (6). All parameters seem to improve in comparison with pre-operative status, but remain unrestored in comparison with normal individuals (4, 5). Studies show that altered hip biomechanics start early on from mild-to-moderate hip osteoarthritis (OA) for the majority of the gait parameters described above, with hip adduction moment differentiating the severe stages of hip OA (7, 8). Minor deficits have been spotted between surgical approaches, but their clinical relevance remains unknown (5, 9). Asymmetrical loading seems to exist even during activities of daily life, such as sit-to-stand, and all above-mentioned residual biomechanical deficits may affect adjacent joints’ health, implant longevity, and patient satisfaction (4, 5, 10).

Over time, the field of biomechanics has seen major advancements in methodology. Traditional gait laboratories using optical 2D motion capture and force plates have provided valuable data over the years; however, more recently, wearable inertial measurement units (IMUs), 3D motion capture systems, and dynamic fluoroscopy have enabled *in vivo* analysis in real-world or under high-precision conditions, allowing researchers to assess subtle functional impairments or compensations that may go undetected in routine clinical follow-up (11, 12, 13). Evolution in clinical gait analysis during the past 50 years has led to easier, faster, and more massive data collection on gait biomechanics, with refined algorithms and musculoskeletal models providing more accurate results with clinical relevance (14). Biomechanics allows for an assessment of movement, capturing abnormalities before they even reach a clinically significant level, with increased sensitivity during various activities of daily life, allowing for clinical correlations while modeling even allows for prediction in various scenarios (14, 15).

A quantitative report of all biomechanical modalities, including all activities of daily life, is currently missing in the literature, as current systematic reviews focus on isolated time points or isolated biomechanical parameters of gait, missing important correlations and valuable tools (e.g. dual fluoroscopy, EMG, isokinetic) (4, 5, 6, 9, 16). Current systematic reviews also fail to capture surgical parameters, apart from surgical approach, and how they influence biomechanics, while specific biomechanical methods and how they might influence outcomes measure (e.g. IMUs) are also not accounted for (4, 5, 6, 9, 16). The objective of this systematic review is to synthesize evidence on *in vivo* biomechanical changes post-THA, with a focus on the impact of surgical techniques, anatomical restoration, and patient demographics on gait and joint function during activities of daily life. Comparative studies with the pre-operative status, contralateral limb, and healthy population will be analyzed. All biomechanical assessment tools will be included (3D motion analysis, force platforms, EMG, isokinetic dynamometer, dual fluoroscopy), with the only exception being of IMUs, handheld dynamometers, and instrumented soles due to non-standardized protocols during testing according to the literature (high-quality data during our data synthesis remain a priority).

## Methods

### Search strategy

A systematic review was conducted following the PRISMA 2020 guidelines and was prospectively registered in PROSPERO (CRD42024550457). Two independent reviewers (VG and IS) performed screening, data extraction, and risk of bias assessment, whereas a third reviewer (SX) resolved conflicts by consensus. We searched MEDLINE (via PubMed), Scopus, PEDro, Web of Science, Embase, Cochrane Library, and SPORTDiscus (via EBSCOhost) for studies reporting *in vivo* biomechanical outcomes following primary THA in adult humans with no date restrictions. The search strategy used was ‘((((hip replacement) OR (hip arthroplasty)) OR (hip)) AND (((((((((gait) OR (walk*)) OR (run)) OR (running)) OR (jump*)) OR (squat*)) OR (sit)) OR (stand)) OR (function))) AND ((((((((((((((((((biomechanic*) OR (kinematic)) OR (kinetic)) OR (torque)) OR (power)) OR (electromyography)) OR (emg)) OR (isokinet*)) OR (strength)) OR (grf)) OR (ground reaction force)) OR (force)) OR (moment)) OR (fatigue)) OR (endurance)) OR (acceleration)) OR (activation)) OR (contraction))’ in MEDLINE and adapted accordingly for each search database. The reference list of all articles included in the final review was analyzed for potentially eligible articles. For conflicting results, a thorough discussion was conducted to reach a consensus. In cases where this was not possible, an experienced third reviewer (SX) resolved any conflict.

#### Inclusion criteria

- Human studies evaluating post-operative *in vivo* biomechanics after THA.
- Use of objective tools (e.g. motion capture, force plates, fluoroscopy).
- Report of spatiotemporal movement parameters or joint kinematics or kinetics.
- Comparison with the control group (healthy limb or healthy population or pre-operative status).
- Primary hip osteoarthritis and DDH I indication for the operation.

#### Exclusion criteria

- Non-human studies.
- Isolated low-reliability tools (handheld dynamometers, instrumented soles, IMUs).
- Post-operative physical therapy intervention.
- Reviews, editorials, or case reports.
- Non-English articles.

### Data extraction and synthesis

Two reviewers (VG and IS) independently extracted data on the study design, study purpose/intervention, patient populations, study protocol, surgical approach and materials, follow-up period, assessment methods (activity and tools used), biomechanical outcomes, and study conclusions. Custom spread sheets were used to extract the above-mentioned qualitative and quantitative data. A narrative synthesis approach was employed, categorizing findings into thematic areas, such as gait speed, joint kinematics, and the impact of surgical approach, to account for the methodological diversity among included studies. For studies with closed access, an attempt was made to obtain full text via university subscriptions or communication with the author. All 50 full texts were acquired and analyzed. Bias assessment was performed using the Newcastle–Ottawa Score (NOS) for cohort and case–control studies (total score of 9, low risk: 8–9; moderate: 5–7; high: ≤4) and the RoB2 tool for randomized trials.

## Results

### Study selection process

**Figure 1:**
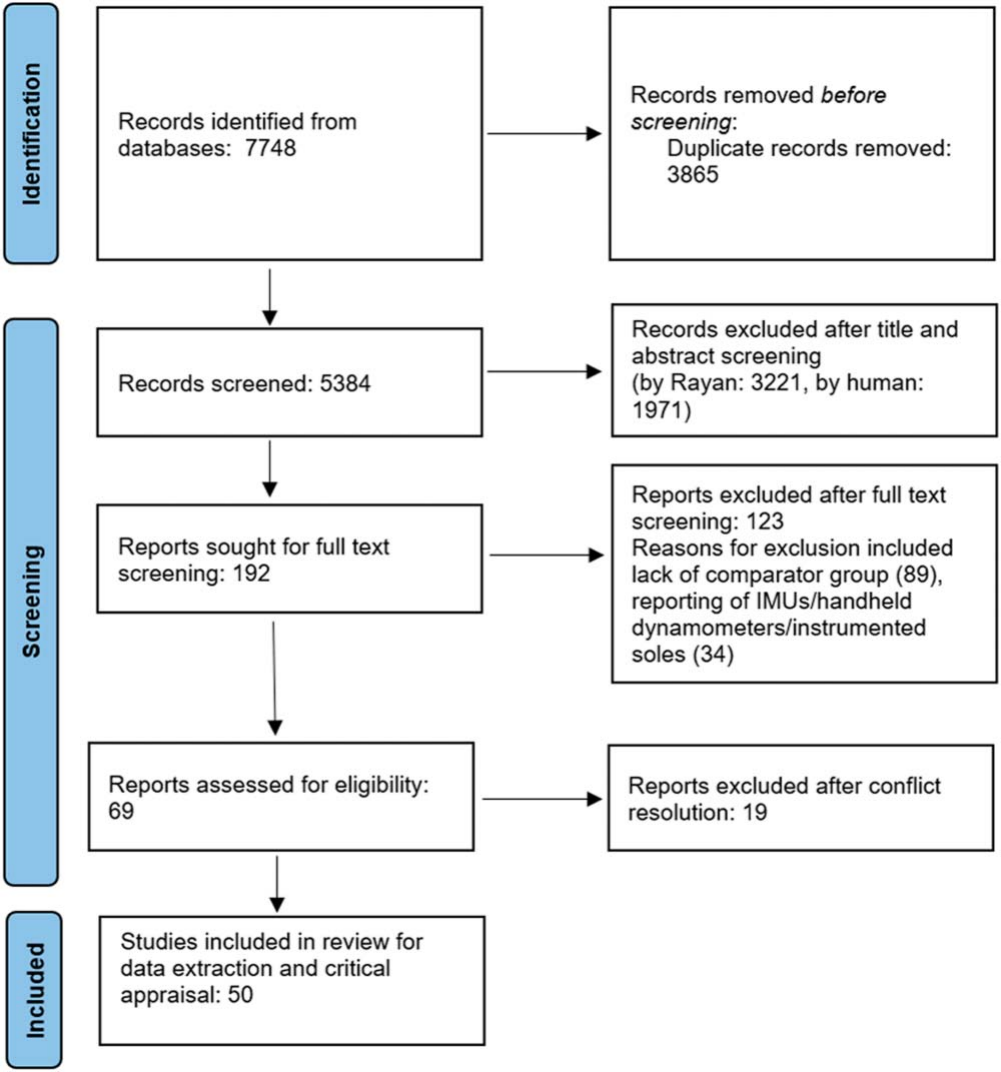
Flow chart of study selection according to the PRISMA 2020 guidelines for new systematic reviews of databases and registries. Fifty studies were included in this qualitative analysis.

In total, 7,748 articles were included. The titles and abstracts of the articles were uploaded to a web-based software platform for systematic reviews with integrated AI tools (Rayyan) for efficient screening. After the removal of duplicates, 5,384 articles were screened for title and abstract. Abstract and title screening declined articles irrelevant to the topic, reporting no biomechanical parameters or including no comparison group, resulting in 192 articles. The full text of these articles was uploaded to the platform. After full-text screening, 69 articles remained. Conflicts were resolved through discussion, focusing on the clarity of reported biomechanical outcomes and the robustness of control group comparisons. An experienced reviewer (SX) was consulted to resolve disagreements, and 50 studies were finally included in this study (Fig. 1).

### Study characteristics

This systematic review included 50 studies, with a median total sample per study of 39 participants (range: 10–243) and an approximate aggregate of ∼3,100 participants across all rows; 17 studies explicitly included healthy control cohorts, while the rest included a comparison of the operated limb with the healthy limb. The study designs were predominantly prospective cohort or cross-sectional comparisons, with 6 of the studies reporting a randomized controlled trial study design. Most studies were categorized as having low or moderate risk of bias (see Supplementary Table 1 and the section in the Supplementary materials given at the end of this article and Table 1). The majority of the studies scored high for selection and exposure but lacked comparability with a healthy control group or did not adjust for BMI and age. Outcome measurements were assessed as low risk for almost all studies due to the implementation of standardized protocols and biomechanical tools. Randomized trials were assessed with some concerns, mainly due to inadequate randomization protocols.

**Table 1:** Bias assessment for the six randomized controlled trials according to the RoB2 tool.

	Zügner *et al.* (56)	Rosenlund *et al.* (53)	Catelli *et al.* (59)	Weber *et al.* (61)	Esposito *et al.* (50)	Cankaya *et al.* (39)
Randomization process	Some concerns	Some concerns	Some concerns	Some concerns	Some concerns	Some concerns
Deviations from intended interventions	Low	Low	Low	Low	Some concerns	Low
Missing outcome data	Some concerns	Low	Low	Low	Low	Low
Measurement of the outcome	Low	Low	Low	Low	Low	Low
Selection of the reported result	Some concerns	Some concerns	Some concerns	Some concerns	Some concerns	Some concerns
Overall risk of bias	Some concerns	Some concerns	Some concerns	Some concerns	Some concerns	Some concerns

Few studies have reported the implants used, with Stryker and DePuy constituting the majority. One study examined the effect of dual-mobility implants, and two studies compared large femoral heads. The surgical approach was specified as a single approach in 35 studies (posterior = 16; lateral = 11; anterior = 8), comparisons (e.g. lateral vs posterior, or three-arm comparisons) were reported in 11 studies, and no approach was reported in four studies. The primary functional task was level walking (36 studies; 72%), with additional assessments including sit-to-stand (4), quiet standing (4), deep squat (2), car ingress/egress (1), multidirectional walking (forward/backward/lateral; 1), timed up-and-go (1), crutch walking (1), and stair ascent/descent (1). Biomechanical evaluation was done by the most commonly used 3D motion capture system (35 studies) and force plates (35 studies); complementary modalities include surface electromyography (EMG) (5), isokinetic dynamometry (4), dual fluoroscopy (4), and instrumented treadmill (1). The follow-up timing clustered at 6 months (16 studies) and 12 months (16 studies), with earlier assessments at 3 months (16), 6 weeks (5), and 2 months (19). Early (≤3 months) assessments were present in 15 studies and late (≥12 months) assessments in 23 studies, with >12-month follow-up explicitly reported in seven studies. Studies using IMUs were excluded due to their varied methodology and potential for inconsistent data capture compared to laboratory-based biomechanical tools. A detailed presentation of the studies in each category is provided in the following. All studies are presented in Supplementary Table 2.

### Gait analysis findings: THA vs control *(healthy or contralateral)*

#### Spatiotemporal outcomes

Most studies reported significant improvements in basic *spatiotemporal* parameters, such as gait speed, stride length, and cadence post-operatively; however, the values remained below those of the control group even at 1-year post-operatively (17, 18). Despite the improvement in stride length and cadence at 1-year compared to pre-operative values, they remained asymmetrical in the early recovery phases (19, 20, 21).

#### Kinematic outcomes

Kinematic analysis entails joint segment motion in space, without regard to forces. Joint angles, range of motion, pelvic tilt, and hip rotation were used to describe limb or joint movement. Kinematic parameters showed a general improvement, with deficits persisting mainly in the frontal and transverse regions. Patients who underwent THA retained lower peak hip extension, abduction, and ROM even two years post-operatively due to less anterior pelvic tilt (22). Furthermore, increased in-phase coordination of the pelvis and thigh (pelvis-thigh working as a unit) was observed in the THA population, suggesting another possible explanation for the decreased ROM (22). However, variations exist, and each patient should be treated on a personalized basis, as Langley *et al.* proved in a supplementary study that high achievers do exist among THA patients and can even reach kinematic outcomes similar to those of the control groups (23). Leijendekkers *et al.* showed that patients with mild DDH may show increased lateral trunk flexion post-op (17). Van Drongelen *et al.* also demonstrated that hip flexion–extension and pelvic tilt are the most important prognostic factors for gait normalization after THA (24). Pincheira emphasized hip abduction impairment during the early post-operative phase (20). Rathod *et al.* and Petis *et al.* identified the posterior approach and tendon releases as predisposing factor for internal/external rotation deficits (25, 26). Mazzoli *et al.* also showed hip extension deficits as the rest of the studies, however, correlating worse outcomes post-operatively with increased patient age (27). Regarding the rest of the joints of the operated limb, a study showed that their ROM increased as a compensatory mechanism for reduced hip ROM (28).

#### Kinetic outcomes

Kinetic data refer to the forces and moments during movement. They are described by the ground reaction forces, joint moments, torques, and power generation. Kinetic outcomes did not recover as reliably as kinematic parameters, with most studies recording deficits in joint moment and power generation. Van Drogel *et al.* presented in 2019 that kinetic parameters are restored in THA patients compared to the control group, but varus alignment of the femoral prosthesis and other radiological parameters are correlated with higher joint moments (21, 29). Stief *et al.* also conducted a high-level study, indicating that THA does not restore joint loading in the hip and knee, as reduced hip extension–abduction led to greater adduction moments in the hip and knee and possibly knee osteoarthritis (22, 30). Da Cunha *et al.* found that Vitamin D levels pre-operatively are correlated with reduced peak power generation, an observation also possibly correlated with the proposed high functioning cluster of THA patients proposed by van Drongel *et al.* and Naili *et al.* (9, 16, 18, 23, 24, 31). Lalevee *et al.* suggested lower vertical ground reaction forces and hip moment arms at 1 year post-operatively, even with a minimally invasive anterolateral approach (32). Aqil *et al.*, using kinetic data from force plates only, also showed that ground reaction forces remain higher than pre-operatively even at 1-year follow-up, possibly leading to osteoarthritis of adjacent joints (33).

#### Electromyography (EMG) studies and isokinetics

Five studies included the EMG evaluation of patients. One study showed decreased maximal isometric contraction of the gluteus medius, maximus, and TFL, suggesting gluteal muscle damage at 13–17 months post-operatively despite using the minimally invasive anterior approach (32, 34). Another study at 13 months post-operatively compared patients of the lateral and posterior approaches to healthy patients and found no difference in isokinetic torque but higher muscle activation amplitudes as the previous group, suggesting possible muscle damage (35). Other studies also confirmed that gait and EMG characteristics are not normalized in THA patients, whereas a study concentrating on isokinetic muscle strength and gait pattern using the Hardinge approach six months post-operatively showed no statistically significant changes (36, 37). A study utilizing superficial EMG showed preservation or even restoration of muscle contraction post-operatively; results either correlated with the anterior approach or with the use of a superficial EMG device (38). Finally, Cankaya *et al.* isolated the isokinetic performance after posterior and anterolateral THA and found no difference between the two groups (39). From the above-mentioned studies, we conclude that EMG is a more sensitive test for recording muscle damage after THA and increased EMG may be detected using the direct lateral approach.

### Functional tasks

Sit-to-stand, stair ascend/descend, standing still, crutch walking, and entrance into the car were among the everyday tasks examined in the studies. A 2022 study examined the entrance in a car, showing that the operated limb shows less ROM and strength and produces less power, compensated by power production in other joints, especially when the pivoting limb is operated, which could damage the other joints (40). A study integrating the walking test and instrumented time-up-and-go (iTUG) test with IMUs showed that deficits in the TUG test were more evident than deficits during walking, and the TUG test could be used to differentiate between high achievers and patients struggling after THA (41). A 2020 study recording sit-to-stand with the VICON system showed that both pre-operative and post-operative patients presented a contralateral and forward shift of the center of mass, reducing hip and knee extension moments, and thus, uploading the affected joint (42). Miura *et al.* also performed two studies in 2018, showing impairments in load distribution in THA patients post-operatively even after 1-year, a possible goal of rehabilitation (43, 44). In 2015, Queen *et al.* also found side-to-side differences indicative of joint unloading during stair descend-ascend pre- and post-operatively (45). Komiyama *et al.* used a model after CT imaging and assessed kinematics during squatting, finding that THA increased hip ROM, allowed for more anterior pelvic tilt during squatting, and thus, no prosthetic impingement (46). All authors showed persistent deficits in moments and power in the affected limb post-operatively and proposed that these deficits should be addressed by physical therapy post-operatively (40, 41, 42, 43, 44, 45, 46). The bipedal stance was the only activity found to normalize loading after THA, showing that THA might improve biomechanics in comparison with the arthritic state, but physical therapy might be needed to incorporate these results into everyday life (47). Although other studies found increased ground reaction forces and impaired center of pressure at more than 1-year post-op, measurements taken with eyes closed were more accurate (48, 49). Finally, the crutch-walking technique during the early post-operative period was shown to influence gait normalization later, with the correct elbow-extended technique being crucial (50).

### Surgical parameters

#### Impact of the surgical approach

Another critical aspect influencing post-THA biomechanics, as our review highlights, is the choice of surgical approach. Among the surgical approaches presented in the current studies are posterior, posterolateral, direct lateral, modified Hardinge, and anterolateral approaches. No difference was found between the lateral and posterior approaches 3 months post-operatively (51). Another study comparing the anterior, lateral, and posterior approaches pre-operatively and at 6 and 12 weeks post-operatively showed that despite similar temporal characteristics, multiple kinematic and kinetic variables differed significantly (25). The same study showed an increased lateral trunk lean and pelvic tilt in the lateral approach, with no differences across abduction moments (possibly study error), whereas external rotation moments were increased with the anterior and posterior approaches (25). A study involved direct lateral, posterior, and anterolateral approaches at 1-year post-operatively, with all groups presenting gait asymmetries post-operatively (52). Another study comparing anterior, lateral, and posterior approach at 1-year showed spatiotemporal biomechanics close to normal only for the anterior approach and showed that hip offset differences of more or less than 5 mm do not affect biomechanics (21). Rathod *et al.*, on the other hand, found no differences comparing the posterior and anterior approaches at 6 months and 1-year, rather found a decrease in external/internal rotation of the hip by the posterior approach (26). In a comparison between the direct lateral and posterior approaches, the posterior approach was found to have greater hip abductor and flexor strength at 12 months (53). Nishimura *et al.* compared anterolateral and direct lateral approaches at nine and 28 weeks and recorded no gait differences and only lower pain scores for the anterolateral group at nine weeks (54). Finally, Robbins *et al.*, in 2020, recorded no differences between the lateral and posterior approaches in terms of gait analysis and isometric torques (35). No consensus was reached between the studies, with minor advantages being recorded for the anterior approach. No consensus has also been reached regarding the effect short external rotators release during THA (55).

#### Implants

One study used a short, femoral-preserving stem, which showed no difference from the standard stem (56). Jensen *et al.* studied the use of large femoral heads (50 mm vs 28/32 mm) and showed that patients receiving large femoral heads displayed less improvement of the gait deviation index over time (57). However, this is in contrast with the results of Stolarczyk *et al.* who noticed that large femoral heads (36 mm vs 28/32 mm) lead to better results in terms of gait biomechanics, possibly explained by the different sizes of the large heads between the two studies (58). Finally, a study comparing dual mobility (DM) with single bearing (SB) at nine months showed that DM implants present functional deficits inherent to implant design and should be used with caution in the active population (59). Similar results were obtained for a series of DM implants by Martz *et al.,* presenting great outcomes but with a slight deficit compared to the literature on SB implants (60). Finally, a study on femur-first computer-assisted THA showed no advantage over conventional THA, apart from an improved hip flexion angle (61). Surgical technique during implantation of the components also has a great, although smaller than surgical approach, impact on post-operative biomechanics, and geometrical restoration of the joint (including femoral offset, varus/valgus alignment, hip center of rotation, and leg length discrepancy) should all be aimed to restore during THA (21, 29, 62).

#### Dual fluoroscopy

Dual-fluoroscopy studies have consistently shown that static radiographs can misrepresent the *in vivo* THA mechanics during functional tasks. In level walking, anatomically ‘well-placed’ cups demonstrated, on average, 10.1° greater functional anteversion and 16.0° lower functional inclination than native hips, placing the acetabular orientation outside the Lewinnek safe zone for >50% of the gait cycle and even during quiet standing, underscoring the influence of spinopelvic dynamics on functional alignment (63). During strenuous tasks, such as step-up and single-leg stance, implanted hips showed a mean 3.4° increase in internal rotation compared to the contralateral side, with this asymmetry strongly associated with component geometry (greater femoral/cup anteversion and anterior joint-center translation), highlighting the kinematic consequences of implant positioning (64). Across the normal, pre-THA degenerative, and post-THA cohorts, dual-fluoroscopy tracking revealed that implanted hips had the least separation, compression, and sliding during gait (degenerative: sliding up to 6.9 mm; normal: up to 1.75 mm), yet edge-loading contact patterns were still observed post-THA, indicating subtle micro-instability and contact mechanics despite reduced macro-separation (65). Finally, treadmill DFIS analysis in unilateral THA documented an average 5.1° increase in internal rotation on the operated side during gait, which correlated with a linear combination of higher cup anteversion, medial cup translation, and leg lengthening, directly linking alignment restoration to rotational symmetry (66). Collectively, these studies highlight the limitations of static imaging, which underestimates the functional behavior of the components, while traditional kinematic analysis methods also fail to highlight marginal differences.

### Long-term follow-up

Short-term follow-up has been extensively analyzed in the above-mentioned studies, with follow-ups of up to 1-year post-operatively consisting the majority of the studies. Few studies have included long-term follow-up due to the complex nature of biomechanical measurements. Harada *et al.* showed that even high-achievers after THA still demonstrate reduced power production and compensation in adjacent joints at 24–205 months of follow-up (40). Langley *et al.*, on the other hand, showed that a cluster of patients after THA achieves normalization of gait characteristics; however, the cluster seems to not include DDH patients (19, 23). Other studies also showed that gait deficits persist for two or more years after THA (29, 32, 56, 58).

## Discussion

According to our study and other systematic reviews, THA is associated with reliable improvements in objective gait performance within weeks to months after surgery. Despite these gains and the relief of pain, patients often do not achieve the functional benchmarks of healthy individuals at the one-year mark, suggesting an incomplete recovery of function (4). Pooled analyses confirm initial gains in walking speed, stride/step length, and hip range of motion by 6–12 weeks. However, significant deficits persist at 12 months relative to healthy controls in key metrics, such as walking speed, stride length, single-limb support, and sagittal hip ROM (peak hip extension) (4, 22, 23, 24, 25, 26, 27, 28).

Across surgical approaches, meta-analytic syntheses report minimal and inconsistent results between-approach differences in spatiotemporal, kinematic, and kinetic variables at early (≤3 months) and late (≥6–12 months) follow-up, suggesting that approach choice per se has a limited influence on gait mechanics (5). When significant approach effects are detected, effect sizes are small, and their clinical meaning remains uncertain, reinforcing that restoration quality and patient factors likely dominate post-operative mechanics. Direct comparisons of anterior approaches further indicate only modest early advantages (for example, small improvements in gait speed and peak hip flexion within three months for direct anterior vs anterolateral approaches), with convergence thereafter, underscoring the transient nature of most approach-related differences (9).

Kinematic recovery is uneven, with sagittal plane excursions improving earlier than frontal/transverse control, which often remains constrained and contributes to asymmetry and compensatory trunk/pelvic strategies during stance (4). It seems that the compensatory trunk lateral flexion adapted from early on during OA remains hardwired in the patient’s gait and is not restored to healthy standards, even though improvement in comparison with pre-op status is noted (7, 8). Kinetic recovery lags in comparison with kinematics, and meta-level synthesis consistently highlights a reduced hip abduction moment compared with controls after THA, a deficit also evident during stair negotiation or ascend/descend from a chair, where demands on frontal plane control are greater (10, 16). Abductor strength shows progressive improvement through 6–24 months, but may not fully normalize, aligning with observed kinetic deficits and supporting targeted strengthening beyond symptom resolution (6). Recent clinical studies similarly reported early post-operative gains in abductor power after THA, reinforcing the modifiability of this impairment with structured recovery and repair integrity (67). Exercise-based rehabilitation programs improve balance and gait performance measures post-THA, supporting the integration of progressive hip abductor/extensor loading and task-specific retraining into standard care, which could be especially helpful for full recovery of the osteoarthritic gait after THA (68).

Beyond straight-line, self-selected walking and higher–demand activities of daily living (ADLs), such as stair ascent/descent, sit-to-stand, and car ingress/egress – unmask larger residual limitations in hip moments and power, indicating the need to assess and train capacity under ecologically valid conditions (16). Heterogeneity in marker sets, modeling conventions, walking speeds, and outcome definitions remains a major barrier to pooling across studies, which mirrors previous reviews’ calls for standardized biomechanical reporting at fixed time points (5). Functional imaging with dual fluoroscopy adds an important dimension by demonstrating that acetabular components considered ‘well-positioned’ on static radiographs can assume orientations outside traditional safe-zone boundaries during walking and even quiet standing, driven by spinopelvic dynamics (66). These dynamic departures support a shift from solely anatomical targets to functional alignment concepts (for example, combined anteversion ‘functional safe zones’) to better predict stability and *in vivo* mechanics (69). Contemporary reviews of cup positioning similarly question traditional reliance on historical safe zones, emphasizing patient-specific functional orientation and pelvic mobility (70).

### Instrumented-implant (‘telemetric THA’) studies

Telemetric, instrumented THA stems – embedding strain gauges and a radio-frequency transmitter in the femoral neck – provide the current gold standard, *in vivo* measurements of the 3-component hip contact forces and 3-component moments during real activities of daily living (ADLs) (71). Across classic and contemporary Charité (OrthoLoad) cohorts, level walking typically produces peak resultant hip contact forces of ∼2.4–3.0 × body weight (BW), with early work reporting ∼238% of BW and later standardized datasets indicating ∼2.9–3.0 × BW (72, 73). Loads increase with task demand: stair ascent/descent exceeds level walking and real (unwarned) stumbling can transiently reach >8 × BW, underscoring the need to consider rare but extreme events in design and early rehab (72, 74). Using 10 subjects and nine frequent/demanding ADLs, Bergmann *et al.* standardized time-varying reference load profiles (‘AVER75’ and ‘HIGH100’) and showed that current ISO stem tests substantially under-represent true *in vivo* amplitudes/directions – arguing for multi-directional, activity-specific test spectra (72). Beyond forces, these implants quantify friction moments: during walking, friction declines ∼47% from 3 to 12 months post-op (a likely ‘running-in’ and lubrication effect), while many every day or rehab tasks (e.g. sustained single-leg stance, certain stretching, vibration training) can generate friction moments greater than the ∼4 Nm threshold associated with cup fixation risk – supporting cautious progression of early one-legged and sustained-load activities (75, 76). Task-specific guidance emerging from *in vivo* data includes most basic physiotherapy and many gym/aerobic exercises that load the hip at or below walking, but selected maneuvers – especially single-limb variants or board-assisted aerobics – elevate peak forces/torques and may be deferred or supervised early post-op; conversely, aquatic exercise reduces joint forces by ∼36–55% versus land-based equivalents (with caveats at high velocities or with resistive fins) (77, 78). Footwear also seems to have a major impact, with heel-drop shoes seeming increasing the loads acting on the hip, in contrast to barefoot walking or flat shoes, which share the loads on the adjacent joint of the foot, ankle, and knee (79). Gluteal muscle damage after THA should also be a goal of rehabilitation, as it leads to higher joint reaction forces (80). Collectively, telemetric THA datasets (publicly released via OrthoLoad) serve as the ground truth to validate musculoskeletal models and inform patient-specific planning, with validated models reproducing *in vivo* peak-phase magnitudes within ∼8–12% for walking and one-leg stance (81). THA has been an increasingly popular and successful operation, leading to more active patients of lower age and higher expectations. Traditional rehabilitation guidelines have failed to successfully guide return-to-activity, with much heterogenicity, which *in vivo* studies with telemetric hip implants can help shed light into. It is obvious that activities once considered safe can yield high forces, with cycling, gym, and aquatic exercises being one of the few safe options for the early post-op period (82). Despite small cohorts utilizing only specific implants, these studies uniquely anchor our understanding of real-world THA loading, refine preclinical testing, and guide rehabilitation/activity recommendations, in contrast to what is known currently (72). Direct measurement of forces via instrumented hip implants seems to be the gold standard for kinetic data nowadays, providing more reliable data than inverse kinematic methods, such as 3D motion analysis and force plates (83, 84).

### Wearable inertial measurement units (IMUs)

Based on our protocol, IMU-only studies were excluded. However, we considered hybrid setups (IMUs synchronized with validated video/optical systems) because contemporary evidence indicates that IMUs can yield clinically useful gait metrics when their accuracy is benchmarked against reference systems (85). IMUs demonstrate excellent concurrent validity for mean spatiotemporal parameters (e.g. speed, cadence, step/stride length) during walking, supporting their use in ambulatory follow-up. However, numerous factors can affect their reliability, such as fluctuation of sensor performance during their lifetime or poor factory quality control, all of which are attributed to the lack of strict protocols for IMU measurements (85, 86). For segment/joint kinematics, several comparative studies against optoelectronic motion capture reported typical errors in the 3–8° RMSE range with high waveform agreement, particularly in the sagittal plane during functional movements (87, 88). Recent systematic reviews further conclude that lower-limb kinematic estimates from IMUs are promising but method-dependent, reinforcing the need for transparent calibration and validation against gold-standard references (89, 90). Methodologically, error sources include magnetometer disturbance, soft-tissue artifact from on-skin placement, and integration drift, which can degrade orientation estimates and especially impact transverse-plane hip rotation if not mitigated with robust sensor fusion and task-specific calibration (86, 87, 88, 89, 90, 91). Comparative validations of commercial and research IMU systems confirm good agreement for lower-limb angles when protocols control these factors, although accuracy varies across joints/axes and worsens with highly dynamic motion (92, 93). In the THA context, early work has shown that IMU-derived features can distinguish THA gait from controls and support mobile feedback, illustrating the clinical utility for longitudinal monitoring when paired with appropriate validation (94).

In summary, wearable IMUs offer scalable, real-world assessment of post-THA recovery, particularly for spatiotemporal outcomes, while hybrid IMU + video/optical configurations provide the measurement fidelity needed for research-grade kinematics in our review. Adherence to current ISB recommendations on sensor placement, calibration, and accuracy reporting versus skeletal motion remains essential for interpretable datasets (85, 86).

### Limitations

Study heterogeneity (tasks, timing, modeling methods), modest sample sizes, and limited confounder control (adjustment for BMI, age, weight, etc.) limited our study. Because of our restricted inclusion–exclusion criteria to ensure the high quality of the included studies (exclusion of IMUs and inclusion of only studies including comparison with healthy limbs or subjects), we were not able to reach a sufficient number of studies to perform a meta-analysis. Only the subcategory lateral vs posterior approach showed four studies at three months and five studies at 1-year, but lack of data in some papers (despite request from the authors) and data heterogeneity (in terms of follow-up and outcomes) did not allow for further meta-analysis. These limitations likely attenuate detectable between-group differences and preclude meta-analyses.

Key learning points

- Persistent deficits: despite pain relief, patients retain kinetic deficits (reduced power and hip extension) > 1 year post-op, often compensating with the lumbar spine.
- Static vs dynamic: dual-fluoroscopy reveals that cups ‘safe’ on static X-rays often edge-load during gait; functional alignment targets may be superior to Lewinnek’s safe zone.
- True joint loads: telemetric implants show that ‘benign’ activities, such as stumbling or single-leg stance, can generate forces >3–8 × body weight.
- Surgical impact: surgical approach (direct anterior vs posterior) has a minimal long-term effect on gait; restoring femoral offset and anteversion is more critical than the incision used.

## Conclusion

The current body of evidence suggests that despite achieving significant functional gains, THA does not fully restore kinetic and control-related deficits one year after the procedure. As the surgical approach demonstrates only minor and inconsistent effects, a focus on three key areas is needed to improve outcomes: rehabilitation of frontal-plane control and strength, assessment under high-demand ADLs, and meticulous attention to functional component orientation. While the reliability of wearable IMUs remains a challenge, they hold promise for monitoring patient progress. Moreover, due to substantial errors inherent in inverse dynamics, the wider use of highly accurate instrumented implants is crucial for advancing our understanding of THA biomechanics and especially kinetics, whereas data from such studies should be highly regarded during post-operative instructions in THA patients. We recommend that future research efforts standardize biomechanical data reporting, integrate functional alignment principles, leverage instrumented implants, and evaluate targeted rehabilitation interventions through well-designed comparative trials.

## Supplementary materials



## ICMJE Statement of Interest

The authors declare that there is no conflict of interest that could be perceived as prejudicing the impartiality of the work reported.

## Funding Statement

This work did not receive any specific grant from any funding agency in the public, commercial, or not-for-profit sector.

## Ethical review

Ethical Review Committee Statement was not required for the presented study. The presented work was performed at the Physical Therapy Department, University of Patras, Patras, Greece.
