# Supplementary material for: In vivo biomechanics after total hip arthroplasty: a state-of-the-art systematic review
Source: EFORT Open Rev. 2026 Jul 1;11(7):758–69. doi: 10.1530/EOR-2026-0003 (PMC13326882; doi:10.1530/EOR-2026-0003)
Supplement: Supplementary file 1 [file supplementary_materials.pdf]

# Supplementary Materials

Supplementary Table 1: Bias assessment for the 44 non-randomized studies according to Newcastle-Ottawa Score.

| **Author** | **S: Representativeness** | **S: Non-exposed Cohort** | **S: Ascertainment** | **S: Outcome Not Present at Start** | **O: Confounding Factors** | **O: Design/Analysis** | **O: Assessment** | **O: Follow-up Length** | **O: Adequacy of Follow-up** | **Total Score (0-9)** | **Risk of Bias** |
|---|---|---|---|---|---|---|---|---|---|---|---|
| **Jensen et al., 2015 [1]** | 1 | 1 | 1 | 1 | 1 | 1 | 1 | 1 | 0 | 8 | **Low** |
| **Harada et al., 2022 [2]** | 1 | 1 | 1 | 0 | 1 | 1 | 1 | 1 | 0 | 7 | **Low** |
| **Ippolito et al., 2021** | 1 | 1 | 1 | 1 | 0 | 0 | 1 | 1 | 1 | 7 | **Low** |

| | | | | | | | | | | | |
|---|---|---|---|---|---|---|---|---|---|---|---|
| **[3]** | | | | | | | | | | | |
| **Zeni et al., 2018 [4]** | 1 | 1 | 1 | 0 | 0 | 0 | 1 | 0 | 1 | 6 | **Moderate** |
| **Lin et al., 2022 [5]** | 1 | 1 | 1 | 0 | 1 | 1 | 1 | 1 | 0 | 7 | **Low** |
| **Langley et al., 2023 (Gait & Posture) [6]** | 1 | 1 | 1 | 1 | 0 | 0 | 1 | 1 | 1 | 7 | **Low** |
| **Stolarczyk et al., 2022 [7]** | 1 | 1 | 1 | 0 | 1 | 0 | 1 | 1 | 0 | 6 | **Moderate** |
| **Petis et al., 2018 (Can J Surg) [8]** | 1 | 1 | 1 | 1 | 0 | 0 | 1 | 0 | 1 | 6 | **Moderate** |
| **Langley et al., 2023 (Hip Int.) [9]** | 1 | 1 | 1 | 1 | 0 | 0 | 1 | 1 | 1 | 8 | **Low** |
| **Leijendekkers et al., 2018 [10]** | 1 | 1 | 1 | 1 | 1 | 0 | 1 | 1 | 1 | 8 | **Low** |
| **Queen et al., 2014** | 1 | 0 | 1 | 0 | 0 | 0 | 1 | 1 | 1 | 5 | **Moder** |

| | | | | | | | | | | | |
|---|---|---|---|---|---|---|---|---|---|---|---|
| **[11]** | | | | | | | | | | | **ate** |
| **Pincheira et al., 2019 [12]** | 1 | 1 | 1 | 1 | 0 | 0 | 1 | 0 | 1 | 6 | **Moderate** |
| **Gasparutto et al., 2021 [13]** | 1 | 1 | 1 | 1 | 0 | 0 | 1 | 0 | 1 | 6 | **Moderate** |
| **Naili et al., 2019 [14]** | 1 | 1 | 1 | 1 | 1 | 0 | 1 | 1 | 1 | 8 | **Low** |
| **Ryan et al., 2024 [15]** | 1 | 1 | 1 | 1 | 1 | 0 | 0 | 1 | 1 | 7 | **Low** |
| **da Cunha et al., 2016 [16]** | 1 | 0 | 1 | 1 | 0 | 0 | 1 | 0 | 1 | 5 | **Moderate** |
| **Esbjörnsson & Naili, 2020 [17]** | 1 | 1 | 1 | 1 | 1 | 0 | 1 | 1 | 1 | 8 | **Low** |
| **Esbjörnsson et al., 2021 [18]** | 1 | 0 | 1 | 1 | 0 | 0 | 1 | 1 | 1 | 6 | **Moderate** |
| **van Drongelen et** | 1 | 1 | 1 | 1 | 0 | 0 | 1 | 1 | 1 | 7 | **Low** |

| | | | | | | | | | | | |
|---|---|---|---|---|---|---|---|---|---|---|---|
| **al., 2019** [19] | | | | | | | | | | | |
| **Petis et al., 2018 (Hip Int.)** [20] | 1 | 1 | 1 | 1 | 0 | 0 | 1 | 0 | 1 | 6 | **Moderate** |
| **Rathod et al., 2014** [21] | 1 | 1 | 1 | 1 | 0 | 0 | 1 | 1 | 1 | 7 | **Low** |
| **Queen et al., 2015** [22] | 1 | 0 | 1 | 1 | 0 | 0 | 1 | 1 | 1 | 6 | **Moderate** |
| **Martz et al., 2016** [23] | 1 | 1 | 1 | 1 | 0 | 0 | 1 | 1 | 1 | 7 | **Low** |
| **van Drongelen et al., 2023** [24] | 1 | 0 | 1 | 1 | 0 | 0 | 1 | 1 | 1 | 6 | **Moderate** |
| **Stief et al., 2018** [25] | 1 | 1 | 1 | 1 | 1 | 0 | 1 | 1 | 1 | 8 | **Low** |
| **van Drongelen et al., 2021** [26] | 1 | 1 | 1 | 1 | 0 | 0 | 1 | 1 | 1 | 7 | **Low** |
| **Lalevée et al., 2023** [27] | 1 | 1 | 1 | 1 | 0 | 0 | 1 | 1 | 1 | 7 | **Low** |

| | | | | | | | | | | | |
|---|---|---|---|---|---|---|---|---|---|---|---|
| **Martinez et al., 2022 [28]** | 1 | 1 | 1 | 1 | 0 | 0 | 1 | 1 | 1 | 7 | **Low** |
| **Kopeć et al., 2015 [29]** | 1 | 0 | 1 | 1 | 0 | 0 | 1 | 1 | 1 | 6 | **Moderate** |
| **Robbins et al., 2020 [30]** | 1 | 1 | 1 | 1 | 0 | 0 | 1 | 1 | 1 | 7 | **Low** |
| **Çakmak et al., 2024 [31]** | 1 | 0 | 1 | 1 | 0 | 0 | 1 | 1 | 1 | 6 | **Moderate** |
| **Miura et al., 2018 [32]** | 1 | 0 | 1 | 1 | 0 | 0 | 1 | 1 | 0 | 5 | **Moderate** |
| **Aqil et al., 2016 [33]** | 1 | 1 | 1 | 1 | 1 | 1 | 1 | 1 | 1 | 9 | **Low** |
| **Pop et al., 2018 [34]** | 1 | 1 | 1 | 0 | 1 | 0 | 1 | 1 | 1 | 7 | **Low** |
| **Chang et al., 2015 [35]** | 1 | 1 | 1 | 0 | 1 | 0 | 1 | 1 | 0 | 4 | **High** |
| **Miura et al., 2018 [36]** | 1 | 0 | 1 | 0 | 0 | 0 | 1 | 1 | 1 | 7 | **Low** |

| | | | | | | | | | | | |
|---|---|---|---|---|---|---|---|---|---|---|---|
| **Nishimura et al., 2016 [37]** | 1 | 1 | 1 | 0 | 1 | 0 | 1 | 1 | 0 | 6 | **Moderate** |
| **Komiyama et al., 2018 [38]** | 1 | 0 | 1 | 0 | 0 | 0 | 1 | 1 | 1 | 5 | **Moderate** |
| **Mazzoli et al., 2017 [39]** | 1 | 0 | 1 | 0 | 0 | 0 | 1 | 1 | 0 | 4 | **High** |
| **Kolářová et al., 2020 [40]** | 1 | 0 | 1 | 0 | 0 | 0 | 1 | 1 | 0 | 4 | **High** |
| **Tsai et al., 2015 [41]** | 1 | 0 | 1 | 0 | 0 | 0 | 1 | 1 | 1 | 6 | **Moderate** |
| **Zheng et al., 2021 [42]** | 1 | 0 | 1 | 0 | 0 | 0 | 1 | 1 | 1 | 5 | **Moderate** |
| **Dimitriou et al., 2015 [43]** | 1 | 0 | 1 | 0 | 0 | 0 | 1 | 1 | 1 | 5 | **Moderate** |
| **DeCook et al., 2019 [44]** | 1 | 0 | 1 | 0 | 0 | 0 | 1 | 1 | 1 | 5 | **Moderate** |

Supplementary Table 2: Presentation of the 50 studies

| Study (year) | Comparison/Design | Follow-up (timing) | Activity | Sample (n) | Surgical approach | Biomechanical methods | Key findings (concise) |
|---|---|---|---|---|---|---|---|
| Jensen et al. (2015) [1] | Preoperative vs postoperative; Lateral vs Posterior; Large-head vs standard-head THA | Preoperative 2-months 6-months | Level walking | Conventional THA n=19; Large-Head THA n=16; healthy controls n=20 | Lateral, Posterior | 3D motion capture, Force plates | Patients who received 50 mm LHA showed less improvement in GDI compared to patients who received conventional 28/32 mm THA. |
| Harada et al. (2022) [2] | THA vs healthy controls | L-THA: 68.9 ± 59.5 months R-THA: 44.2 ± 32.2 months | Car ingress/egress | Left THA n=20 Right THA n=20 healthy controls n=30 | Posterior | 3D motion capture, Force plates | Even high-functioning unilateral THA patients have limited RoM and power production in the surgical hip; they compensate for this loss by producing power in |

| | | | | | | | |
|---|---|---|---|---|---|---|---|
| | | | | | | | other joints, especially when the surgical side is the pivot limb |
| Langley et al. (2023) [6] | THA vs healthy controls | 21 ± 16 months | Level walking | THA n=10; healthy controls n=10 | Posterior | 3D motion capture, Force plates | Patients after THA have significantly lower peak hip extension and RoM, which is the result of significantly less anterior tilt of the pelvis, which limits the thigh RoM. |
| Ippolito et al. (2021) [3] | Preoperative vs postoperative; THA vs healthy controls; Anterior vs Lateral | Preoperative 3 months 6 months | Level walking (forward, backward, | THA n=14; healthy controls n=14 | Anterior | 3D motion capture, Surface EMG | Hip and gait function during several locomotor tasks improved after surgery while simultaneously preserving or restoring |

| | | | | | | | |
|---|---|---|---|---|---|---|---|
| | | | lateral) | | | | muscle activation around the hip joint. |
| Zeni et al. (2018) [4] | Preoperative vs postoperative; Lateral vs Posterior | 3 months | Level walking | 45;Lateral n=23; Posterior n=22 | Posterior, Lateral | 3D motion capture, Force plates | The surgical approach did not affect the biomechanical outcomes three months after arthroplasty. |
| Lin et al. (2022) [5] | THA vs healthy controls | 12 months | Level walking | THA n=12; hip OA n=12; healthy controls n=12 | Posterior | 3D motion capture, Force plates, Isokinetic dynamometer | One year after hip arthroplasty, muscle strength recovered sufficiently for normal walking but not to avoid falling in risky situations. |
| Stolarczyk et al. (2022) [7] | THA vs healthy controls | At least 3.5 years (mean: 44 months) | Level walking | Large femoral head (36mm) n=15; Standard femoral | Anterolateral | 3D motion capture | The use of larger femoral heads during THR provides better results in |

| | | | | | | | |
|---|---|---|---|---|---|---|---|
| | | | | head (28-32mm) n=15; healthy healthy controls n=15 | | | terms of the gait pattern. |
| Petis et al. (2018) [8] | Preoperative vs postoperative; Anterior vs Lateral vs Posterior | Preoperative 6 weeks 12 weeks | Level walking | n=30 (10 anterior; 10 posterior; 10 lateral approach) | Anterior, Lateral, Posterior | 3D motion capture, Force plates | Temporal gait parameters were similar following THA in all approaches. |
| Langley et al. (2023) [9] | THA vs healthy controls | 22 ± 16 months | Level walking | THA n=10; healthy controls n=11 | Posterior | 3D motion capture, Force plates | The presence of high-functioning THA patients who display comparable sagittal plane hip kinematics to controls suggests that these patients achieve normative function and |

| | | | | | | | |
|---|---|---|---|---|---|---|---|
| | | | | | | | challenges the conclusions of previous group-level analysis. |
| Leijendekkers et al. (2018) [10] | Operated limb vs contralateral limb; THA vs healthy controls | 49.8 ± 54.1 months post-THA | Level walking | 6 women with DDH treated with unilateral THA; 8 healthy controls women matched for age and sex | Posterior | 3D motion capture, Force plates | In patients with DDH treated with IBG, modest asymmetries in gait kinematics and kinetics were observed, with the exception of a substantial asymmetry of trunk lateral flexion. |
| Queen et al. (2014) [11] | THA vs healthy controls; Anterolateral vs Lateral vs Posterior | 12 months | Level walking | 35 with primary unilateral THA (12 Lateral; 18 P; 11 AL approaches) | Anterolateral, Lateral, Posterior | 3D motion capture, Force plates | No significant differences were observed among the different surgical approach groups for any |

| | | | | | | | |
|---|---|---|---|---|---|---|---|
| | | | | | | | of the study variables. |
| Pincheira et al. (2019) [12] | Preoperative vs postoperative; THA vs healthy controls; Lateral vs Posterior | , 3 weeks and 12 weeks | Level walking | 11 Lateral THA n=11 Posterior THA | Lateral, Posterior | 3D motion capture | The surgical approach does not affect hip kinematics at the early postoperative stage after arthroplasty. |
| Gasparutto et al. (2021) [13] | Preoperative vs postoperative; THA vs healthy controls; Lateral vs Posterior | 6 months | Timed Up-and-Go (TUG) | THA n=71 [Rottinger (n=38); Mini-posterior (n=29); Lateral (n=4)] healthy controls n=52 | Posterior, Lateral | 3D motion capture | Patients with THA presented deficits in all four phases of the TUG test when compared to the control group before and six months after surgery, even though their motor function improved in all phases. |
| Zügner et | THA vs healthy | 24 months | Level | THA n=44; CFP | Lateral | 3D motion | The use of a CFP stem |

| | | | | | | | |
|---|---|---|---|---|---|---|---|
| al. (2022) [45] | controls; Short curved stem vs conventional stem THA | | walking | stem n=22; Corail stem n=22; healthy controls n=66 | | capture, Force plates | did not significantly influence any of the gait parameters studied when compared to a standard stem; however, both stems studied were associated with gait deviations when compared to controls. |
| Naili et al. (2019) [37] | Preoperative vs postoperative; THA vs healthy controls | 12 months | Level walking | 34 with unilateral symptomatic primary hip OA; 25 healthy healthy healthy controls | Anterolateral | 3D motion capture, Force plates | The findings of this study suggest that objectively measured improvements in performance-based function and gait are not in line with patient-reported functional |

| | | | | | | | |
|---|---|---|---|---|---|---|---|
| | | | | | | | improvements, highlighting the importance of using both subjective and objective methods for evaluating gait. |
| Ryan et al. (2024) [15] | THA vs healthy controls; Anterior vs Lateral vs Posterior | 12 months | Level walking | ANT n=16; LAT n=17; POS n=14 | Anterior, Lateral, Posterior | Force plates | The anterior and lateral approaches led to more normal gait biomechanics in sagittal and frontal kinematics, respectively, but only the ANT group exhibited spatiotemporal gait parameters within normal ranges. |
| da Cunha | Preoperative vs | 3 months | Level | 93 completed | Lateral | 3D motion | The 25(OH)D levels |

| | | | | | | | |
|---|---|---|---|---|---|---|---|
| et al. (2016) [16] | postoperative | | walking | spatiotemporal and kinematic analyses n=66 completed the kinetic analyses | | capture, Force plates | correlated with changes in peak extension and peak power generation. |
| Esbjörnsson et al. (2020) [17] | Preoperative vs postoperative; THA vs healthy controls | 12 months | Sit-to-stand | hip OA (pre-THA) n=28; Post-THA n=28; healthy controls n=21 | Anterolateral | 3D motion capture | By quantifying the center-of-mass trajectory during five sit-to-stand test performances, functional movement compensation can be detected and evaluated over time. |
| Esbjörnsson et al. (2021) [18] | Preoperative vs postoperative | Preoperative, 12 months | Level walking | n=65 | Posterior | 3D motion capture, Force plates | Geometrical restoration during THA affects postoperative gait pattern and, in addition to known |

| | | | | | | | |
|---|---|---|---|---|---|---|---|
| | | | | | | | factors such as FO, height of hip rotation center, and leg length discrepancy, FNA must also be taken into consideration. |
| van Drongelen et al. (2019) [19] | Operated limb vs contralateral limb; THA vs healthy controls | Preoperative 1 year postoperatively | Level walking | 22 unilateral hip OA; healthy controls n=15 | Lateral | 3D motion capture, Force plates | In our patient group, the joint moments after total hip replacement did not differ from those of healthy controls, whereas radiological leg alignment parameters changed significantly after total hip replacement. |

| | | | | | | | |
|---|---|---|---|---|---|---|---|
| Petis et al. (2018) [20] | Preoperative vs postoperative; Anterior vs Posterior | Preoperative 6 weeks 12 weeks | Level walking | Anterior release n=5; Anterior no release n=5; Posterior n=5 | Anterior, Posterior | 3D motion capture, Force plates | Releasing the short external rotators during the anterior approach did not cause significant temporal and kinematic changes after THA. |
| Rathod et al. (2014) [21] | Preoperative vs postoperative; Anterior vs Posterior | Preoperative 6 months 12 months | Level walking | Anterior n=11; Posterior n=11 | Anterior, Posterior | 3D motion capture, Force plates | A similar recovery pattern was observed for gait parameters in patients who underwent DAA THA and PA THA at 6-months and 1-year postoperatively. |
| Rosenlund et al. (2016) [46] | Preoperative vs postoperative; Lateral vs Posterior | preoperative 3 months 12 months | Level walking | LA n=22; n Posterior n=22 | Lateral, Posterior | 3D motion capture, Force plates | Overall gait function in the posterior approach group did not improve |

| | | | | | | | |
|---|---|---|---|---|---|---|---|
| | | | | | | | more than that in the lateral approach group. |
| Catelli et al. (2017) [47] | Preoperative vs postoperative; THA vs healthy controls; Dual mobility vs single-bearing THA | Preoperative 9 months | Deep squat | Dual mobility n=12; Single-bearing n=12; healthy healthy healthy controls n=12 | Anterior | 3D motion capture | Neither of the THA implant groups were able to return pelvis and hip kinematics to the level of CTRLs. |
| Queen et al. (2015) [22] | Preoperative vs postoperative | Preoperative 6 weeks 12 months | Stair ascent/descent | N= 42 (lateral= 13, posterior= 19, anterolateral= 10) | Lateral | 3D motion capture, Force plates | Abnormal movement patterns on the surgical side increase demands on other joints and can lead to permanent joint damage. |
| Martz et al. (2017) [23] | Preoperative vs postoperative | Preoperative 6 months | Level walking | ttinger n=38; Moore n=32; | Posterior | 3D motion capture | The combination of the Röttinger approach with |

| | | | | | | | |
|---|---|---|---|---|---|---|---|
| | | | | healthy controls n=61 | | | a dual-mobility cup remains a valid choice for primary THA without a functional advantage at midterm. |
| Weber et al. (2016) [48] | Preoperative vs postoperative; Computer-assisted vs conventional THA | Preoperative6 months 12 months | Level walking | CAS FF n=28; CON n=32 | Not reported | 3D motion capture, Force plates | While all parameters significantly increased over the follow-up period in both groups, no significant differences were found between them at any given time point. |
| van Drongelen et al. | Preoperative vs postoperative; THA vs healthy controls | Preoperative 12 months | Quiet standing | unilateral hip OA piatients n=43 (24M/19F) and | Not reported | 3D motion capture, Force plates | Preoperatively, the non-affected limb carried 10% more body weight |

| | | | | | | | |
|---|---|---|---|---|---|---|---|
| (2023) [24] | | | | n=17 healthy controls (8M/9F).All n=30-80 y.o. and BMI<35 | | | than the affected limb when standing on both legs. |
| Stief et al. (2018) [25] | Preoperative vs postoperative; Operated limb vs contralateral limb; THA vs healthy controls | Preoperative2 2.8 ± 2.3 months | Level walking | 15 (6F/9M) with hip OA underwent THA matched with n=15 healthy conrols | Lateral | 3D motion capture, Force plates | In conclusion, THR for patients with unilateral hip OA does not restore the abnormal loading of the hip and knee joints to normal. |
| van Drongelen et al. (2021) [26] | Preoperative vs postoperative; THA vs healthy controls | PreoperativeAt least 12 months postoperatively | Level walking | 51 symptomatic hip OA . 3 did not fit cluster and were left out finally. 48 finally | Lateral | Force plates | Hip flexion-extension and pelvic tilt were highlighted as the most important variables in the 3D gait analysis to |

| | | | | | | | |
|---|---|---|---|---|---|---|---|
| | | | | | | | discriminate these patients. |
| Lalevee et al. (2023) [27] | THA MIALA patients vs asymptomatic subjects Gait and muscle function after THA via the minimally invasive anterolateral approach (MIALA) | 15.5 months postoperative | Level walking | THA n=16; healthy controls n=15 asymptomatic | Anterolatera l | Force plates, Surface EMG | Gait deficits persisted beyond one year after THA with MIALA. |
| Martinez et al. (2022) [28] | THA vs healthy controls | 15.9 ± 3.1 months | Level walking | MIAA n=15; Asymptomatic n=12 | Anterior | 3D motion capture, Force plates, Surface EMG | Persistent Gait Deficits after THA with MIAA. |
| Kopeć et | Operated limb vs | Preoperative | Level | n=16 | Lateral | 3D motion | Space and time gait |

| | | | | | | | |
|---|---|---|---|---|---|---|---|
| al. (2015) [29] | contralateral limb | 6-8 months postoperative | walking | | | capture, Force plates, Surface EMG | parameters with regard to OP. |
| Robbins et al. (2020) [30] | THA vs healthy controls; Lateral vs Posterior | 13 months (range 11–18 months for lateral, 11–15 months for posterior THA) | Level walking | 63 participants (21 lateral THA; 21 posterior THA; 21 healthy controls) | Lateral, Posterior | 3D motion capture, Force plates, Isokinetic dynamometer, Surface EMG | There were few differences in the gait and isometric torque between the lateral and posterior THA groups. |
| Çakmak et al. (2024) [31] | Preoperative vs postoperative; Operated limb vs contralateral limb; THA vs healthy controls | Preoperatively 3 months 6 months | Level walking | 27 hip OA (14 women; 13 men) | Lateral | Force plates, Isokinetic dynamometer | Adequate muscle strength and physiological gait pattern, similar to the preoperative status, can be achieved 6 months |

| | | | | | | | |
|---|---|---|---|---|---|---|---|
| | | | | | | | postoperatively. |
| Dimitriou et al. (2015) [43] | Operated limb vs contralateral limb; THA vs healthy controls | 12.4 ± 4.6 months | Level walking | THA n=15 | Posterior | 3D motion capture, Dual fluoroscopy | There were significant in vivo kinematic differences in the THA hips compared to the contralateral native hip during stair climbing. |
| DeCook et al. (2020) [44] | Preoperative vs postoperative; THA vs healthy controls | Preoperativeo perative 6 weeks 12 weeks | Level walking | 10 healthy healthy controls n=10 with hip OA (7 THA) | Anterior | | Trends in degenerative hips show more abnormal hip kinematics, leading to higher articulating surface forces and stresses within the acetabulum. |
| Tsai et al. (2015) [41] | Operated limb vs contralateral limb; | 10.6 ± 4.6 months | Level walking | 19 unilateral THA | Posterior | 3D motion capture, Dual | Total hip arthroplasty patients compensated for |

| | | | | | | | |
|---|---|---|---|---|---|---|---|
| | THA vs healthy controls | | | | | fluoroscopy | changes in hip geometry by altering dynamic movement during gait. |
| Zheng et al. (2021) [42] | Operated limb vs contralateral limb; THA vs healthy controls | 11.5 months (± 4.2, range 6.8– 22.6) | Quiet standing | 17 (4M/13F) | Posterior | 3D motion capture, Dual fluoroscopy | In conclusion, this study quantified continuous acetabular orientation during weight-bearing functional activities. |
| Cankaya et al. (2023) [49] | Preoperative vs postoperative; Anterolateral vs Posterior | Preoperative 6 months 12 months | Level walking | Anterolateral n=24 Posterior n=24 | Posterior | Isokinetic dynamometer | Although there is concern about potential abductor muscle damaging during ALA, the results of this randomised controlled study demonstrated that ALA can produce similar |

| | | | | | | | |
|---|---|---|---|---|---|---|---|
| | | | | | | | isokinetic performance and functional outcome to PA at 6 and 12 |
| Chang et al. (2015) [35] | Preoperative vs postoperative | Preoperative<br>2 weeks<br>6 weeks<br>3 months<br>6 months<br>1 year | Quiet standing | n=23 | Lateral | Force plates | The recovery of postural stability and functional capacity in one year duration fluctuated, and no consistent improvement tendency was found. |
| Miura et al. (2018) [32] | THA vs healthy controls | 12 months | Sit-to-stand | THA n=28; healthy controls n=16 | Posterior | Force plates | Persisting asymmetry in lower limb loading after THA compared to the typical right-left in healthy adults without lower limb pain or |

| | | | | | | | |
|---|---|---|---|---|---|---|---|
| | | | | | | | sensorimotor impairment. |
| Miura et al. (2018) [36] | Preoperative vs postoperative | Preoperative<br>1 month<br>2 months<br>3 months<br>6 months<br>12 months | Sit-to-stand | 158 THA | Posterior | Force plates | Longitudinal and dynamic assessment of Op loading |
| Aqil et al. (2016) [33] | Preoperative vs postoperative; Operated limb vs contralateral limb; THA vs healthy controls | Preoperative<br>13 months | Sit-to-stand | Postoperative n=17; healthy healthy healthy controls n=17 | Posterior | Force plates, Instrumented treadmill | Although gait becomes more symmetrical, patients still experience higher peak loads than the matched controls. |
| Esposito et al. (2018) [50] | Preoperative vs postoperative; Operated limb vs | 2 months | Level walking | EF n=15; EE n=15 | Not reported | Force plates | The crutch setup influenced gait after the patients started to walk |

| | | | | | | | |
|---|---|---|---|---|---|---|---|
| | contralateral limb; THA vs healthy controls; Crutch setup EF vs EE | | | | | | unassisted. |
| Pop et al. (2018) [34] | THA vs healthy controls | between 24 and 36 months postoperatively (mean time since surgery: −29,2 months) | Quiet standing | 55 ( | Not reported | Force plates | Static balance parameters in the THR group can still be impaired up to 2-3 years after surgery, compared to the age-matched, asymptomatic control group. |
| Mazzoli et al. (2017) [39] | Preoperative vs postoperative | Preoperative<br>2 weeks<br>6 months<br>12 months | Level walking | 20 elderly THA | Posterior | 3D motion capture | One year from total hip arthroplasty, age affects hip joint limitations and gait recovery more than preoperative passive |

| | | | | | | | |
|---|---|---|---|---|---|---|---|
| | | | | | | | restrictions due to muscle shortening. |
| KolÁŘovÁ et al. (2020) [40] | Preoperative vs postoperative | Preoperative<br>3 months<br>6 months | Level walking | 10 males | Lateral | 3D motion capture | During walking after THR, the sagittal range of motion in the ankle of the unOp. |
| Nishimura et al. (2016) [37] | Preoperative vs postoperative; Anterolateral vs Lateral | Preoperative<br>9 weeks<br>28 weeks | Level walking | Anterolateral n=7; Lateral n=8 | Lateral, Anterolateral | 3D motion capture | Hip pain at 9 weeks after surgery was significantly improved using ALS compared with the DL approach, but no significant differences were found in gait function at 28 weeks after surgery using ALS or DL. |

| | | | | | | | |
|---|---|---|---|---|---|---|---|
| Komiyama et al. (2018) [38] | Preoperative vs postoperative | 43.3 months | Deep squat | | Posterior | Single fluoroscopy 3D-to-2D model from CT scans Computer Simulation | THA increased the range of hip joint motion, and the pelvis tilted anteriorly more after THA, with sufficient liner-to-neck clearance during squatting. |

References


1. Jensen, C.; Penny, J.; Nielsen, D.B.; Overgaard, S.; Holsgaard-Larsen, A. Quantifying Gait Quality in Patients with Large-Head and Conventional Total Hip Arthroplasty--A Prospective Cohort Study. *J Arthroplasty* **2015**, *30*, 2343-2348.e2341., doi:10.1016/j.arth.2015.06.049.
2. Harada, T.; Hamai, S.; Hara, D. Three-dimensional kinematics and kinetics of getting into and out of a car in patients after total hip arthroplasty. *Gait Posture* **2022**, *98*, 305-312., doi:10.1016/j.gaitpost.2022.10.003.
3. Ippolito, G.; Serrao, M.; Conte, C. Direct anterior approach for total hip arthroplasty: Hip biomechanics and muscle activation during three walking tasks. *Clin Biomech (Bristol)* **2021**, *89*, 105454., doi:10.1016/j.clinbiomech.2021.105454.
4. Zeni, J., Jr.; Madara, K.; Witmer, H.; Gerhardt, R.; Rubano, J. The effect of surgical approach on gait mechanics after total hip arthroplasty. *J Electromyogr Kinesiol* **2018**, *38*, 28-33., doi:10.1016/j.jelekin.2017.11.004.
5. Lin, X.; Wu, W.; Weijer, R.H.A. Strong relationship of muscle force and fall efficacy, but not of gait kinematics, with number of falls in the year after Total Hip Arthroplasty for osteoarthritis: An exploratory study. *Clin Biomech (Bristol)* **2022**, *92*, 105551., doi:10.1016/j.clinbiomech.2021.105551.
6. Langley, B.; Whelton, C.; Page, R. Exploring pelvis and thigh movement and coordination patterns during walking in patients after total hip arthroplasty. *Gait Posture* **2023**, *103*, 196-202., doi:10.1016/j.gaitpost.2023.05.023.
7. Stolarczyk, A.; Stolarczyk, M.; Oleksy, Ł. Analysis of biomechanical gait parameters in patients after total hip replacement operated via anterolateral approach depending on size of the femoral head implant: retrospective matched-cohort study. *Arch Orthop Trauma Surg* **2022**, *142*, 4015-4023., doi:10.1007/s00402-021-04264-6.
8. Petis, S.; Howard, J.; Lanting, B.; Jones, I.; Birmingham, T.; Vasarhelyi, E. Comparing the anterior, posterior and lateral approach: gait analysis in total hip arthroplasty. *Can J Surg* **2018**, *61*, 50-57., doi:10.1503/cjs.003217.
9. Langley, B.; Page, R.M.; Whelton, C. Do patients with well-functioning total hip arthroplasty achieve typical sagittal plane hip kinematics? A proof of concept study. *Hip Int* **2023**, *33*, 247-253., doi:10.1177/11207000211044471.
10. Leijendekkers, R.A.; Marra, M.A.; Kolk, S. Gait symmetry and hip strength in women with developmental dysplasia following hip arthroplasty compared to healthy subjects: A cross-sectional study. *PLoS One* **2018**, *13*, e0193487., doi:10.1371/journal.pone.0193487.
11. Queen, R.M.; Appleton, J.S.; Butler, R.J. Total hip arthroplasty surgical approach does not alter postoperative gait mechanics one year after surgery. *PM R* **2014**, *6*, 221-226., doi:10.1016/j.pmrj.2013.09.006.
12. Pincheira, P.A.; De La Maza, E.; Silvestre, R.; Guzmán-Venegas, R.; Becerra, M. Comparison of total hip arthroplasty surgical approaches by Statistical Parametric Mapping. *Clin Biomech (Bristol)* **2019**, *62*, 7-14., doi:10.1016/j.clinbiomech.2018.12.024.

13. Gasparutto, X.; Gueugnon, M.; Laroche, D.; Martz, P.; Hannouche, D.; Armand, S. Which functional tasks present the largest deficits for patients with total hip arthroplasty before and six months after surgery? A study of the timed up-and-go test phases. *PLoS One* **2021**, *16*, e0255037., doi:10.1371/journal.pone.0255037.
14. Naili, J.E.; Hedström, M.; Broström, E.W. Changes of and interrelationships between performance-based function and gait and patient-reported function 1 year after total hip arthroplasty. *J Orthop Traumatol* **2019**, *20*, 14., doi:10.1186/s10195-019-0521-7.
15. Ryan, N.S.; Kowalski, E.; Beaulé, P.E.; Lamontagne, M. The Effect of Surgical Approach and Hip Offset Reconstruction on Gait Biomechanics Following Total Hip Arthroplasty. *J Arthroplasty* **2024**, *39*, 402-408.e401., doi:10.1016/j.arth.2023.08.040.
16. da Cunha, B.M.; Gava, A.D.; de Oliveira, S.B.; de David, A.C.; Dos Santos-Neto, L.L. Vitamin d is related to gait recovery after total hip arthroplasty: A prospective analysis. *Gait Posture* **2016**, *50*, 96-101., doi:10.1016/j.gaitpost.2016.08.014.
17. Esbjörnsson, A.C.; Naili, J.E. Functional movement compensations persist in individuals with hip osteoarthritis performing the five times sit-to-stand test 1 year after total hip arthroplasty. *J Orthop Surg Res* **2020**, *15*, 151., doi:10.1186/s13018-020-01663-0.
18. Esbjörnsson, A.C.; Kiernan, S.; Mattsson, L.; Flivik, G. Geometrical restoration during total hip arthroplasty is related to change in gait pattern - a study based on computed tomography and three-dimensional gait analysis. *BMC Musculoskelet Disord* **2021**, *22*, 369., doi:10.1186/s12891-021-04226-4.
19. van Drongelen, S.; Kaldowski, H.; Tarhan, T.; Assi, A.; Meurer, A.; Stief, F. Are changes in radiological leg alignment and femoral parameters after total hip replacement responsible for joint loading during gait? *BMC Musculoskelet Disord* **2019**, *20*, 526., doi:10.1186/s12891-019-2832-5.
20. Petis, S.M.; Vasarhelyi, E.M.; Howard, J.L.; Lanting, B.A. Gait analysis following release of the short external rotators during an anterior approach for total hip arthroplasty. *Hip Int* **2018**, *28*, 584-590, doi:10.1177/1120700017752514.
21. Rathod, P.A.; Orishimo, K.F.; Kremenic, I.J.; Deshmukh, A.J.; Rodriguez, J.A. Similar improvement in gait parameters following direct anterior & posterior approach total hip arthroplasty. *J Arthroplasty* **2014**, *29*, 1261-1264., doi:10.1016/j.arth.2013.11.021.
22. Queen, R.M.; Attarian, D.E.; Bolognesi, M.P.; Butler, R.J. Bilateral symmetry in lower extremity mechanics during stair ascent and descent following a total hip arthroplasty: a one-year longitudinal study. *Clin Biomech (Bristol)* **2015**, *30*, 53-58., doi:10.1016/j.clinbiomech.2014.11.004.
23. Martz, P.; Bourredjem, A.; Laroche, D. Röttinger approach with dual-mobility cup to improve functional recovery in hip osteoarthritis patients: biomechanical and clinical follow-up. *Int Orthop* **2017**, *41*, 461-467., doi:10.1007/s00264-016-3245-9.
24. van Drongelen, S.; Holder, J.; Stief, F. Lower limb joint loading in patients with unilateral hip osteoarthritis during bipedal stance and the effect of total hip replacement. *Front Bioeng Biotechnol* **2023**, *11*, 1190712., doi:10.3389/fbioe.2023.1190712.
25. Stief, F.; Schmidt, A.; van Drongelen, S. Abnormal loading of the hip and knee joints in unilateral hip osteoarthritis persists two years after total hip replacement. *J Orthop Res. Published online March 14* **2018**, doi:10.1002/jor.23886.

26. van Drongelen, S.; Stetter, B.J.; Böhm, H.; Stief, F.; Stein, T.; Meurer, A. Identification of Patients with Similar Gait Compensating Strategies Due to Unilateral Hip Osteoarthritis and the Effect of Total Hip Replacement: A Secondary Analysis. *J Clin Med* **2021**, *10*, 2167., doi:10.3390/jcm10102167.
27. Lalevée, M.; Martinez, L.; Rey, B. Gait analysis after total hip arthroplasty by direct minimally invasive anterolateral approach: A controlled study. *Orthop Traumatol Surg Res* **2023**, *109*, 103521., doi:10.1016/j.otsr.2022.103521.
28. Martinez, L.; Noé, N.; Beldame, J. Quantitative gait analysis after total hip arthroplasty through a minimally invasive direct anterior approach: A case control study. *Orthop Traumatol Surg Res* **2022**, *108*, 103214., doi:10.1016/j.otsr.2022.103214.
29. Kopeć, K.; Kusz, D.; Sobota, G.; Nowak, K.; Mierzwiński, M.; Nowak, M. Gait analysis in patients after unilateral hip arthroplasty. *Ortop Traumatol Rehabil* **2015**, *17*, 39-50., doi:10.5604/15093492.1143535.
30. Robbins, S.M.; Gomes, S.K.; Huk, O.L.; Zukor, D.J.; Antoniou, J. The Influence of Lateral and Posterior Total Hip Arthroplasty Approaches on Muscle Activation and Joint Mechanics During Gait. *J Arthroplasty* **2020**, *35*, 1891-1899.e1895., doi:10.1016/j.arth.2020.02.037.
31. Çakmak, M.F.; Bayram, S.; Birişik, F. The effects of modified hardinge approach on hip muscle strength in patients with primary hip arthroplasty: a patient evaluation with isokinetic strength test and gait analyses. *Eur J Orthop Surg Traumatol* **2024**, *34*, 1209-1218., doi:10.1007/s00590-023-03778-6.
32. Miura, N.; Tagomori, K.; Ikutomo, H.; Nakagawa, N.; Masuhara, K. Asymmetrical loading during sit-to-stand movement in patients 1 year after total hip arthroplasty. *Clin Biomech (Bristol)* **2018**, *57*, 89-92., doi:10.1016/j.clinbiomech.2018.06.017.
33. Aqil, A.; Wiik, A.; Zanotto, M.; Manning, V.; Masjedi, M.; Cobb, J.P. The Effect of Hip Arthroplasty on Osteoarthritic Gait: A Blinded, Prospective and Controlled Gait Study at Fast Walking Speeds. *J Arthroplasty* **2016**, *31*, 2337-2341., doi:10.1016/j.arth.2016.03.002.
34. Pop, T.; Szymczyk, D.; Majewska, J. The Assessment of Static Balance in Patients after Total Hip Replacement in the Period of 2-3 Years after Surgery. *Biomed Res Int* **2018**, *2018*, 3707254., doi:10.1155/2018/3707254.
35. Chang, C.J.; Lin, N.L.; Lee, M.S.; Chern, J.S. Recovery of Posture Stability at Different Foot Placements in Patients Who Underwent Minimally Invasive Total Hip Arthroplasty: A One-Year Follow-Up Study. *Biomed Res Int* **2015**, *2015*, 463792., doi:10.1155/2015/463792.
36. Miura N, P.T.; Bs, T.K.P.T.; Bs, I.H.P.T.; Nakagawa N, P.T.; PhD, M.K.M.D.; PhD. Leg loading during quiet standing and sit-to-stand movement for one year after total hip arthroplasty. *Physiother Theory Pract* **2018**, *34*, 529-533., doi:10.1080/09593985.2017.1422203.
37. Nishimura, M.; Takahira, N.; Fukushima, K.; Uchiyama, K.; Moriya, M.; Yamamoto, T. Comparison of Gait Motion Including Postoperative Trunk Deflection Between Direct Lateral and Anterolateral Approaches in Supine Total Hip Arthroplasty. *J Arthroplasty* **2016**, *31*, 1603-1608., doi:10.1016/j.arth.2015.12.043.
38. Komiyama, K. Dynamic hip kinematics during squatting before and after total hip arthroplasty." Journal of orthopaedic surgery and research vol. *13,1 162. 3 Jul* **2018**, doi:10.1186/s13018-018-0873-3.

39. Mazzoli, D.; Giannotti, E.; Longhi, M.; Prati, P.; Masiero, S.; Merlo, A. Age explains limited hip extension recovery at one year from total hip arthroplasty. *Clin Biomech (Bristol)* **2017**, *48*, 35-41., doi:10.1016/j.clinbiomech.2017.07.003.
40. KolÁŘovÁ, K.; VodiČka, T.; BozdĚch, M.; Repko, M. 3D kinematic analysis of patients' gait before and after unilateral total hip replacement. *Acta Bioeng Biomech* **2020**, *22*, 165-171.
41. Tsai, T.Y.; Li, J.S.; Dimitriou, D.; Kwon, Y.M. Does component alignment affect gait symmetry in unilateral total hip arthroplasty patients? *Clin Biomech (Bristol)* **2015**, *30*, 802-807., doi:10.1016/j.clinbiomech.2015.06.010.
42. Zheng, N.; Hu, X.; Dimitriou, D.; Dai, K.; Guo, T.; Tsai, T.Y. Well-Placed Acetabular Component Oriented Outside the Safe Zone During Weight-Bearing Daily Activities. *Front Bioeng Biotechnol* **2021**, *9*, 664907., doi:10.3389/fbioe.2021.664907.
43. Dimitriou, D.; Tsai, T.Y.; Li, J.S.; Nam, K.W.; Park, K.K.; Kwon, Y.M. In vivo kinematic evaluation of total hip arthroplasty during stair climbing. *J Orthop Res* **2015**, *33*, 1087-1093., doi:10.1002/jor.22837.
44. DeCook, C.A.; LaCour, M.T.; Nachtrab, J.K.; Khasian, M.; Dessinger, G.M.; Komistek, R.D. In Vivo Determination and Comparison of Total Hip Arthroplasty Kinematics for Normal, Preoperative Degenerative, and Postoperative Implanted Hips. *J Arthroplasty* **2020**, *35*, 588-596., doi:10.1016/j.arth.2019.08.057.
45. Zügner, R.; Tranberg, R.; Kärrholm, J.; Puretic, G.; Mohaddes, M. No difference in gait pattern between a short collum femoris-preserving and a conventional stem: 44 randomised total hip arthroplasty patients stem evaluated after 2 years. *Hip Int* **2022**, *32*, 452-459., doi:10.1177/1120700020967645.
46. Rosenlund, S.; Broeng, L.; Overgaard, S.; Jensen, C.; Holsgaard-Larsen, A. The efficacy of modified direct lateral versus posterior approach on gait function and hip muscle strength after primary total hip arthroplasty at 12months follow-up. *An explorative randomised controlled trial. Clin Biomech (Bristol)* **2016**, *39*, 91-99., doi:10.1016/j.clinbiomech.2016.09.011.
47. Catelli, D.S.; Kowalski, E.; Beaulé, P.E.; Lamontagne, M. Does the Dual-Mobility Hip Prosthesis Produce Better Joint Kinematics During Extreme Hip Flexion Task? *J Arthroplasty* **2017**, *32*, 3206-3212., doi:10.1016/j.arth.2017.04.049.
48. Weber, T.; Dendorfer, S.; Bulstra, S.K.; Grifka, J.; Verkerke, G.J.; Renkawitz, T. Gait six month and one-year after computer assisted Femur First THR vs. *conventional THR. Results of a patient- and observer- blinded randomized controlled trial. Gait Posture* **2016**, *49*, 418-425., doi:10.1016/j.gaitpost.2016.06.035.
49. Cankaya, D.; Inci, F.; Karakuş, D.; Turker, H.B.; Kahve, Y.; Neyisci, C. Isokinetic performance and function are similar after total hip arthroplasty applied with a posterior or anterolateral approach: a randomised controlled trial. *Hip Int* **2023**, *33*, 67-72., doi:10.1177/11207000211012989.
50. Esposito, F.; Freddolini, M.; Marcucci, M.; Latella, L.; Corvi, A. Unassisted quiet standing and walking after crutch usage in patients with total hip replacements: Does crutch length matter? *Gait Posture* **2018**, *64*, 95-100., doi:10.1016/j.gaitpost.2018.06.004.
